# Territorial Cavalry Ambulance

**Published:** 1908-05-02

**Authors:** 


### The Royal Army Medical Corps Section.

### TERRITORIAL CAVALRY AMBULANCE.

One of the most interesting of the experiments in connection with the newly formed Territorial Army is the institution of special field ambulances for the cavalry branch of the Service. The establishment of complete inclusive field ambulances, in lieu of the former bearer companies and field hospitals, involves but slight change in the already existing Volunteer organisation. But the inauguration of special Mounted Brigade Field Ambulances is a departure the development of which will be watched with more than ordinary interest. The method of training and the special equipment suited to the rapid movements of a cavalry brigade are matters upon which there is much diversity of opinion, and the type of vehicle which will conform best to such requirements has not yet been decided, if indeed it has yet been devised.

According to the special Army Order issued on March 18 in connection with the organisation and establishment of the Territorial Force, 56 field ambulances will be formed with a total of 504 officers and 10,682 men. Of these fourteen will be mounted brigade ambulances, with 84 officers and 1,442 men.

Each cavalry ambulance consists of but two sections, as against three in the infantry field ambulance, and provides accommodation for fifty patients, as compared with the infantry accommodation for 150. The establishment is six officers and 103 men, as compared with ten officers and 220 men. A quartermaster is not provided, whereas one is included in the list of the ten officers of the infantry unit. In neither case is there a special transport officer. In both cases, however, the transport *personnel* for vehicles is provided by the units themselves, and not by the Army Service Corps as in the Regular Army, or Army of the first line as it is now termed, and one of the officers of the bearer division will perform the duties of transport officer.

The Mounted Brigade Field Ambulance is divided, in a similar manner to the infantry unit, into bearer and tent divisions and transport, and each section will consist of one-half of the bearer and one-half of the tent division. It may be well to summarise the detail of the establishment as laid down in the new order.

The bearer division consists of two captains or subalterns, four corporals, two buglers, and thirty-four privates, a total of two officers and forty men. The tent division, with accommodation for fifty patients, consists of four officers (lieutenant-colonel, major, and two captains or subalterns), a sergeant-major, seven sergeants, six corporals, and sixteen privates, a total of four officers and thirty men. Of the sergeants two are allocated to nursing duties, two as stewards, two as compounders, and one is supernumerary; of the corporals two are cooks, two pack store-keepers, and two clerks. Ten men are allotted to the nursing department as orderlies, while there are two cooks, two washermen, and two supernumeraries. The transport section includes two sergeants, twenty regular drivers for vehicles, two drivers for spare draught horses, three other spare drivers, and six batmen or servants, a total of thirty-three. Attached to the unit will be two A.S.C. drivers for forage carts, and one permanent Staff sergeant-instructor. This instructor is in addition to the Regular instructors for both transport and medical work at the Territorial school through which the *personnel* will pass.

For annual training nine riding horses are allowed—one for each officer, one for the sergeant-major, and one for each of the transport sergeants. Thirty draught horses are also allowed for vehicles. These vehicles are: Four heavy Mark V. or VI. ambulance wagons, each with four horses; four light Mark I. wagons, each with two horses; two double-horsed general service wagons for baggage; two single-horse forage carts, the drivers being furnished from the Army Service Corps as above indicated.

This complete *personnel*, like that of the other departments of the Medical Service, will be raised not by the County Associations, but by the Director-General of the Army Medical Service, and as soon as each unit is raised it will be handed over to its County Association for administration and maintenance.

The whole organisation has been well conceived, and we hope that these new units will soon be formed and an attempt made to get them speedily into working order. Their efficiency will depend upon the fulfilment of the promise as to vehicles and upon the equipment supplied, which has been woefully deficient in the past. If the starvation process which so hampered the Volunteer force is continued, the whole of the new scheme is foredoomed. The details of equipment we have not dealt with, as they are not included in the special new order referred to. But they must be on a workable scale, so that the various members of the unit may become acquainted with the work which they—on paper—will be in training to perform. That training, however, must not be only on paper. It must be real, actual training if the country is to be asked to rely on the new Territorial Medical Service. In the same way, if the authorities are in earnest in their desire for this efficiency—by this we mean the Treasury authorities: the earnestness of the Director-General is well known—then they must provide the necessary equipment, let the cost be what it may.

